# The constricting effect of reduced coronary artery compliance on the left ventricle is an important cause of reduced diastolic function in patients with coronary heart disease

**DOI:** 10.1186/s12872-022-02809-0

**Published:** 2022-08-17

**Authors:** Liang Lv, Xianghe Ma, Yannan Xu, Qiong Zhang, Shanshan Kan, Xiaoming Chen, Huajin Liu, Hongwei Wang, Changhua Wang, Jiangwei Ma

**Affiliations:** 1 The Third Affiliated Hospital, Southern Medical University or The Third School of Clinical Medicine, Southern Medical University, Guangzhou, Guangdong China; 2 Shanghai Maritime University, Shanghai, China; 3 Anhui University of Science and Technology, Huainan, Anhui China; 4 Department of Cardiology, Fengxian Branch of Shanghai 6th People’s Hospital, Nanfeng Road 6600#, Shanghai, 201400 China; 5 TengZhou City Central People Hospital, Affiliated to Jining Medical University, Jining, Shandong China

**Keywords:** Left ventricular diastolic function, Coronary heart disease, PCI, Stent implantation

## Abstract

**Background:**

Previous studies of left ventricular diastolic function (LVDF) have focused on the decrease in active and passive diastolic function due to ischemic factors but have not investigated if the decrease in compliance of the coronary arteries that bypass the surface of the heart and travel between the myocardium could cause a constricting effect on the ventricular wall like that caused by myocardial fibrosis.

**Methods and Results:**

581 patients diagnosed with coronary heart disease (CHD) were divided into A group (patients are the control group), B group (patients with less than 50% coronary artery stenosis), C group (patients with coronary artery stenosis between 50 and 75%), D group (patients with coronary artery stenosis greater than 75%) according to the degree of coronary stenosis. The diastolic function of the ventricle is reflected by applying the relaxation time constant T value, which refers to the time between peak dp/dt and end-diastolic pressure in the left ventricle. It was concluded that there was a statistical difference in Gensini scores between patients in groups B, C and D (*P* < 0.001). And multiple linear regression analysis showed that T was correlated with Gensini score and C-dp/dtmax (R = 0.711, *P* < 0.001). Grouping according to the site of stent implantation and the number of stents implanted, it was found out that the changes in T values before and after left anterior descending artery (LAD) stent implantation were greater than left circumflex artery (LCX) and right coronary artery (RCA) (*P* < 0.001). And multiple linear regression revealed a correlation between T values and stent length, ventricular stiffness, and C-dp/dtmax (*P* = 0.001).

**Conclusions:**

The decrease in compliance of the coronary arteries bypassing the surface of the heart and travelling between the myocardium would cause a constricting effect on the ventricular wall like that caused by myocardial fibrosis.

**Supplementary Information:**

The online version contains supplementary material available at 10.1186/s12872-022-02809-0.

## Introduction

In recent years, coronary artery atherosclerotic heart disease has caused an increasement in morbidity and mortality rate and is the leading cause of death worldwide [1]. Several studies have shown that coronary artery disease could cause structural remodeling of the left ventricle, adversely affecting left ventricular diastole and myocardial stiffness leading to reduced left ventricular diastole and increased stiffness, which in turn increases cardiac filling pressures and diastolic insufficiency prior to ventricular systolic dysfunction[2, 3]. Left ventricular diastolic dysfunction (LVDD), which occurs in up to 34% of patients with coronary artery disease, is closely associated with myocardial ischemia and has been shown to alter its clinical course [4–6]. Non-obstructive coronary sclerotic plaques are not sufficient to cause myocardial ischemia but can promote alterations in vascular tone function and may also alter LVDF[4, 7], while it has been suggested that preventing the development of atherosclerosis may help to reduce the incidence of LVDD in the diabetic population [8], suggesting that coronary sclerosis may be involved in the development of LVDD independently of myocardial ischemia affecting vascular elasticity. Atherosclerosis of the coronary arteries, which travel on the surface of the heart, and the rich network of capillaries distributed between the myocardium, are essential for the supply of blood to the myocardium. And atherosclerosis of the coronary arteries leads to a decrease in vascular elasticity, which has a constricting effect on the heart like that of myocardial fibrosis on the ventricular wall. The effect theoretically also causes a decrease in myocardial diastolic function. Previous studies have focused on the reduction in active and passive diastolic function due to ischemic factors but less on coronary atherosclerosis.

PCI is currently the choice of treatment for patients with coronary artery disease who have severe stenosis [9]. One study showed that half of patients’ diastolic function improved in 3–4 years after PCI, while diastolic function remained unchanged or worsened in the remaining patients [10]; another study showed an improvement in left ventricular diastolic function 3 months after stenting [11], suggesting that PCI may affect LVDF in patients, but these studies did not further investigate if the reduced coronary artery compliance caused by stenting influences LVDF.

Therefore, it is necessary to investigate if coronary atherosclerosis and the reduced compliance of coronary arteries and small vessels distributed between the myocardium caused by the implantation of stents during PCI could cause a certain constricting effect on the heart and thus affect the diastolic function of the heart, so that the changes in diastolic function in patients undergoing PCI with stents can be investigated and appropriate therapeutic measures can be taken in a timely and rapid manner to delay or prevent the onset of left ventricular diastolic insufficiency.

## Methods

### Study design and patients

**Fig. 1:**
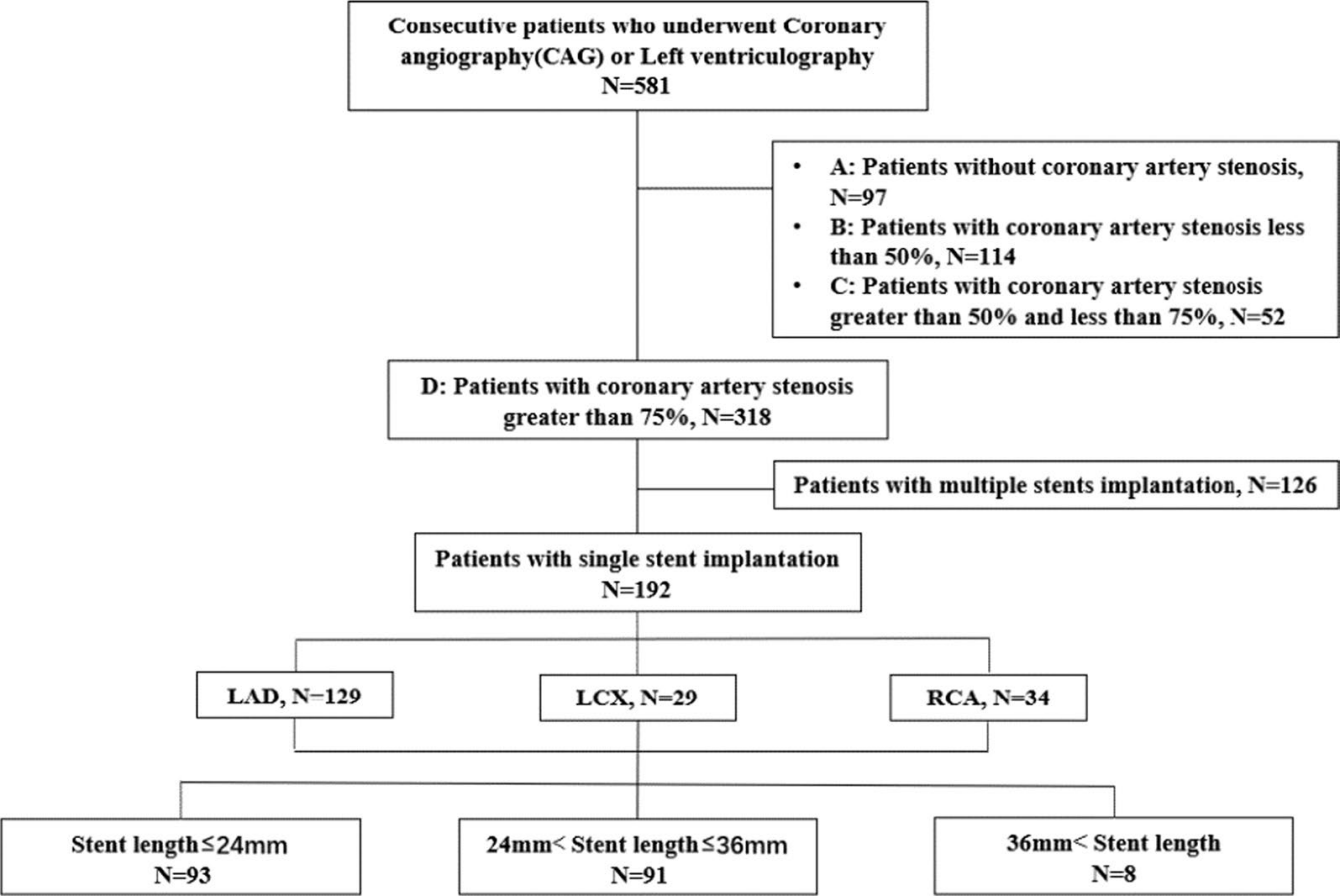
Study design and patients. **a** Control group, **b** coronary artery atherosclerosis group, **c** coronary artery atherosclerotic heart disease group, **d** PCI group

Inclusion subjects: From October 2016 to October 2019, 581 patients diagnosed with CHD in the Department of Cardiology, Fengxian District Central Hospital, of which 318 patients underwent PCI (Fig. 1), were selected under a retrospective study. All enrolled patients signed an informed consent form, and the study was approved by the Ethics Committee (full name: the Shanghai Fengxian District Central Hospital Medical Ethics Committee) (reference number: 2014-KY-06).

Inclusion criteria: (1) the diagnostic criteria for unstable angina referred to the 2007 release of the Chinese Society of Cardiovascular Diseases; (2) Doppler ultrasound examination was performed within 3 days before PCI and 1 year after the repeat imaging, and the left ventricular ejection fraction was greater than 50%; (3) complete clinical data; (4) no history of iodine allergy.

Exclusion criteria: (1) patients with a combined history of acute myocardial infarction or old myocardial infarction; (2) various organic heart diseases such as rheumatic heart valve disease, cardiomyopathy, myocardial amyloidosis, ventricular wall tumor, congenital heart disease and pericardial disease as suggested by cardiac ultrasound; (3) patients with heart failure of New York Heart Association (NYHA) cardiac function class III or above; (4) patients with chronic obstructive pulmonary disease (5) patients with atrial fibrillation; (6) patients with chronic underlying diseases such as severe hepatic or renal insufficiency or severe anemia; (7) patients requiring repeat PCI; and (8) patients with co-infectious diseases.

The 581 patients selected were divided into four groups according to the degree of coronary artery stenosis. Group A patients were the control group. Group B patients had less than 50% coronary artery stenosis. Group C patients had a coronary stenosis between 50 and 75%. Group D patients had a coronary stenosis greater than 75%. And control patients were selected and grouped by an age interval of 10: group 1 (45 ≤ age < 55), group 2 (55 ≤ age < 65), group 3 (65 ≤ age < 75), group 4 (75 ≤ age).

### Coronary angiography and intervention

Coronary angiography and stenting were performed using standard interventional techniques according to the practice guidelines established by the Chinese Society of Interventional Cardiology. Cardiac catheterization and hemodynamic measurements were performed using a large C-wall digital subtraction X-ray system (Model: AXIOM Artis Zee Celling, Device serial number: 147191, Device identification number: 720-939180) and its matching polysomnography. Coronary angiography was performed by an experienced associate chief cardiologist or above, using multiple projections. And two experienced cardiac catheterists quantitatively evaluated the presence or absence of stenosis and vascular stenosis, selected for stent implantation according to the degree of coronary lesion, and collected the length and number of stents in patients with stent implantation. The successful PCI refers to: residual coronary stenosis < 10% after stent implantation, and visualized after angiography Assessment without significant intimal tears, distal embolism, coronary slow flow, occluded side branch occlusion. Gensini scoring method: all patients were scored for each patient's coronary lesion using the modified Gensini score under American Heart Association criteria [12].

### DF measurement and analysis

Left ventriculography: Patients were instructed to assume a right anterior oblique position, photographed at a 30° angle and the contrast agent iodixanol was injected at a certain rate using a high-pressure syringe, with 50 frames/s as the filming speed for coronary angiography in patients with coronary artery disease. Measurements were made according to the playback. The electrocardiogram, left ventricular pressure curve and aortic pressure curve are also recorded simultaneously for at least 5 consecutive cardiac cycles.

Measurement of left ventricular diastolic function indicators: maximum left ventricular filling rate (PFR), maximum rate of left ventricular pressure rise (LV+dp/dtmax) and maximum rate of left ventricular pressure fall (LV-dp/dtmax), systolic + dp/dtmax and diastolic-dp/dtmax, and left ventricular isovolumic diastolic relaxation are measured according to the left ventricular pressure curve using a pressure guidewire system. Time constant of relaxation (T, normal value is < 40 ms) refers to the time between the peak dp/dt and the end-diastolic pressure of the left ventricle.

1 $$T = - {1 \mathord{\left/ {\vphantom {1 A}} \right. \kern-\nulldelimiterspace} A} \quad A = {{Ln\Delta P} \mathord{\left/ {\vphantom {{Ln\Delta P} t}} \right. \kern-\nulldelimiterspace} t}\quad \Delta P = P_{u} - P_{l}$$

(P_u_, Pressure upper limit; P_l_, Pressure lower limit; t, time of pressure changes between P1 and P2).

The inverse of the slope of this line is T, which reflects active left ventricular diastolic function [13–16]. The left ventricular stiffness index (K) is obtained by approximating the Diastolic-Pressure–Volume Curve (DPV) using the data points collected at the end of diastole. The tangent slope of any point on this curve (dP/dV) is called the left ventricular lumen stiffness. And K is the slope of the linear relationship between ventricular stiffness and ventricular pressure, reflecting left ventricular Passive diastolic function [17, 18]. Coronary pressure was measured by end-diastolic coronary volume (CEDV), end-systolic coronary volume (CESV), maximum rate of increase in coronary pressure (C+dp/dtmax). Maximum rate of decrease in coronary pressure(C-dp/dtmax) were measured using pressure guidewires as described above. The coronary angiography (CAG) was reviewed in all patients from 9 months to 1 year after stenting, and indicators reflecting cardiac volumes, size and left ventricular systolic and diastolic function were collected.

Coronary 64-layer spiral CT: A retrospective cardiac gated spiral scan was performed using a Philips Brilliance 64-layer CT machine to select an appropriate scan protocol. After the scan, 20 (5%, 10%, 15%, 20%, 25%, 30%, 35%, 40%, 45%, 50%, 55%, 60%, 65%, 70%, 75%, 80%, 85%, 95%, 100%) ECGs were obtained by automatic offline reconstruction using 5% intervals with a reconstruction layer thickness of 1.5 mm and a spacing of 1.0 mm, Kemal value (convolution value) B26f. (90%, 95%, 100%) cardiac cycles. The reconstructed images were transferred to the Syngo workstation, and the 5–100% full-phase images were transferred to the Circulation software for analysis of cardiac function. 21 time-phase reconstructions were transferred to the post-processing workstation, where two experienced image physicians were selected to perform post-processing and calculate left ventricular end-diastolic volume (LVEDV), left ventricular end-systolic volume (LVESV), and left ventricular systolic volume (LVESV). End-diastolic volume (LVEDV), left ventricular systolic volume (LVESV), maximum rate of left ventricular pressure rise (LV+dp/dtmax), maximum rate of left ventricular pressure fall (LV-dp/dtmax), maximum left ventricular filling rate (PFR), and left ventricular ejection fraction (LVEF).

### Statistical analysis

Baseline demographic data and clinical variables were summarized with continuous variables and expressed as mean ± standard deviation or median with interquartile range. Categorical data were expressed as percentages and number of events. ANOVA or Kruskal–Wallis non-parametric tests for continuous variables as applicable, and χ^2^ tests for categorical data were used to compare if there were differences in LVDF among groups. All analyses used SPSS software version 25.0 and figures used GraphPad Prism 8.4.3. The statistical significance of all the analyses was drawn at a 2-sided significance level, which was 0.05.

## Results

**Fig. 2:**
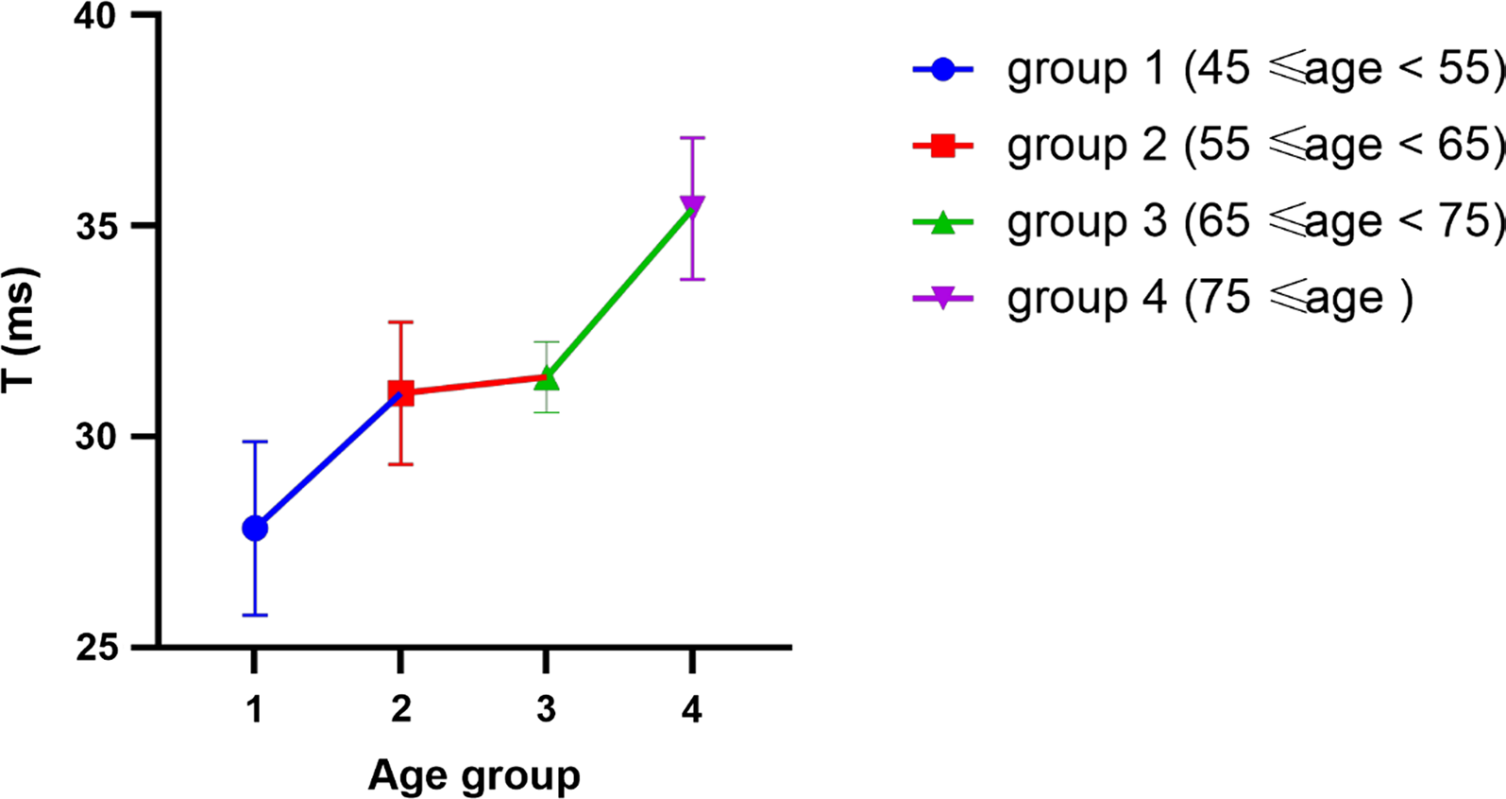
Association between age and T in the A group. The effect of age on diastolic function was explored by grouping patients in the control group by age. A one-way ANOVA on the five groups showed F = 13.893, *p* < 0.001; multiple comparisons showed no statistically significant difference in T-values between groups 2 and 3 (*p* = 0.714); the remaining groups showed statistically significant differences

581 patients were analyzed by a complete data set including LV diastolic function assessed at baseline and follow-up (Table 1). The four groups of patients aged 63.0 ± 9.5 years old and 45.3% of them were male. There was no statistically significant difference in drug use rates of aspirin, atorvastatin, ACEI inhibitors, and beta-blockers among the four groups of patients. Patients in group D had higher blood cholesterol, blood creatinine and BNP compared with those in group A. Control patients were selected and grouped by an age interval of 10: group 1 (45 ≤ age < 55, n = 23), group 2 (55 ≤ age < 65, n = 28), group 3 (65 ≤ age < 75, n = 24), group 4 (75 ≤ age, n = 22) ( A statistically significant difference (F = 14.399, *p* < 0.001) was found that the T values of patients in the different age groups were of statistical values with an increase in the T value of the corresponding diastolic function index as the age of the patients increased (Fig. 2); also, a one-way ANOVA of the Gensini scores of patients in groups B, C and D showed a statistically significant difference (*p* < 0.001). As the degree of coronary artery disease increased, a comparison of DT and LAD measured by echocardiography and T values measured by invasive catheterization, which more accurately reflect the diastolic function of the left ventricle, revealed that patients in groups C and D where the degree of coronary stenosis was greater than 50%, showed a more significant decrease in diastolic function than patients in groups A and B. Patients in group D had poorer diastolic function than those in group C. Correlation analysis of patients in group D with T as the dependent variable showed a correlation between T and Gensini score and C-dp/dtmax (Pearson R = 0.696, − 0.540, *p* < 0.001) and multiple linear regression analysis showed a linear correlation between T and Gensini score and C-dp/dtmax (R = 0.711, B = 0.575, − 0.197, *p* < 0.001). 0.711, B = 0.575, − 0.197, *P* < 0.001). As the degree of coronary atherosclerosis increased, the patient's left ventricular diastolic function also progressively decreased.

**Table 1:** Baseline Characteristics of the Study Patients With CAD Undergoing PCI

	A	B	C	D	*P* value
	(n = 97)	(n = 114)	(n = 52)	(n = 318)	
Male (%)	20 (20.6)	46 (40.4)	33 (63.5)	164 (51.6)	> 0.05
Age, years	61.6 (11.0)	64.6 (11.0)	64.8 (10.2)	62.4 (8.4)	0.027
BSA, m2	1.77 (0.16)	1.78 (0.19)	1.78 (0.37)	1.73 (0.15)	0.085
Smoker (%)	12 (12.4)	28 (24.6)	22 (42.3)	125 (39.3)	> 0.05
Hypertension (%)	25 (25.8)	65 (57.0)	37 (71.2)	200 (62.9)	> 0.05
Diabetes (%)	5 (5.2)	14 (12.3)	11 (21.2)	54 (17.0)	> 0.05
LDL, mmol/L	2.3 (0.6)	2.5(0.6)	2.3 (0.7)	2.3 (0.5)	0.091
HDL, mmol/L	1.1 (0.3)	1.1(0.3)	1.1 (0.3)	1.1 (0.3)	0.611
Serum cholesterol, mmol/L	1.4 (0.4)	1.6 (0.9)	1.7 (1.1)	3.9 (0.8)	> 0.001
Fasting glucose, mmol/L	6.20 (5.16–7.78)	6.05 (5.60–7.00)	6.00 (5.35–7.10)	6.23 (4.53–7.70)	0.601
Serum triglyceride, mmol/L	1.45 (0.72–2.40)	1.22 (0.94–1.77)	1.28 (0.93–1.78)	1.63 (0.93–2.55)	0.097
Serum creatinine, mmol/L	63.5 (49.5–70.5)	65.0 (52.0–71.0)	72.0 (66.0–89.5)	72.9 (56.4–92.3)	< 0.001
BNP, ng/ml	46.4 (23.9–55.1)	46.2 (28.8–71.8)	33.7 (22.7–50.8)	115.0 (23.7–269.8)	< 0.001
Gensini score	0.00^a^	4.0 (2.5–5.0)^b^	14.5 (10.0–17.25)^c^	38.0 (33.2–43.6)^d^	< 0.001
*Angiographic and procedural findings*					
DT, ms	169.1 (61.8)^a^	189.8 (43.4)^a^	217.2 (39.1)^b^	235.7 (79.6)^b^	< 0.001
EF	70.1 (4.8)^a^	69.5 (5.2)^a^	69.3 (4.6)^a^	65.5 (9.1)^b^	< 0.001
LAD, mm	31.8 (2.9)^a^	32.0 (3.3)^a^	35.1 (4.9)^b^	35.1 (6.6)^b^	< 0.001
E/e'	8.4 (0.8)^a^	9.9 (1.6)^b^	11.3 (1.4)^c^	14.6 (2.2)^d^	< 0.001
PFR, ml/s	264.9 (19.1)^a^	242.3 (27.9)^b^	227.2 (21.8)^b^	203.3 (14.8)^c^	< 0.001
T, ms	33.0 (7.6)^a^	34.0 (5.0)^a^	38.5 (6.3)^b^	43.2 (3.8)^c^	< 0.001

^a^Control group, ^b^coronary artery atherosclerosis group, ^c^coronary artery atherosclerotic heart disease group, ^d^PCI group. Values are mean ± standard deviation or n (%). BSA, body surface area; LDL, low-density lipoprotein; HDL, high-density lipoprotein; BNP, brain natriuretic peptide; DT, deceleration time (ms); EF, ejection fraction (%); LAD, anteroposterior diameter of left atrium; PFR, peak early filling rate; T, time constant of relaxation. Each subscript letter denotes a subset of categories whose row proportions do not differ significantly from each other at the .05 level

In order to study left ventricular diastolic function in patients with different degrees of coronary artery stenosis, the enrolled patients were divided into four groups according to the degree of coronary artery stenosis. Group A was the control group, Group B was coronary artery atherosclerosis group, Group C was coronary artery atherosclerotic heart disease group, Group D was PCI group. Basic data were also collected

According to the stent implantation site, patients in Group D were divided into LAD, LCX and RCA groups and further investigated for changes in diastolic function indices preoperatively, immediately postoperatively and 1 year postoperatively. Among the patients with single stents, those with LAD stents got lower preoperative PFR indexes than those with LCX and RCA stents while T, K, LV + dp/dtmax and LV-dp/dtmax indexes were significantly higher than thoses with LCX and RCA stents, and the differences were statistically significant (*P* < 0.001). T, K, LV + dp/dtmax and LV-dp/dtmax were higher than those before stent implantation and the difference was of statistical significance. Diastolic function decreased further, and diastolic function indicators gradually recovered after 1 year postoperatively (Additional file 1: Table S1). The correlation between T and stent length, Gensini score, ventricular stiffness, LV-dp/dtmax, CEDV, CESV and C-dp/dtmax was statistically significant, while multiple linear regression with T as the dependent variable revealed that stent length, C-dp/dtmax and ventricular stiffness were statistically significant (Table 2).

**Table 2:** Correlation of T and clinical parameters

Variables	r	*P* value
Stent length	0.437	< 0.001
Gensini score	0.305	< 0.001
K	0.409	< 0.001
LV-dp/dtmax	− 0.198	0.024
CEDV	− 0.187	0.034
CESV	− 0.252	0.004
C-dp/dtmax	− 0.365	< 0.001
Linear regression-method: stepwise R = 0.556		
Stent length	0.284	0.001
K	0.273	0.001
C-dp/dtmax	− 0.200	0.014
Constant	18.512	0.037

Multiple linearity analysis was performed on data from patients with LAD implantation to investigate the correlation between stent length and the left ventricular diastolic function index T

**Fig. 3:**
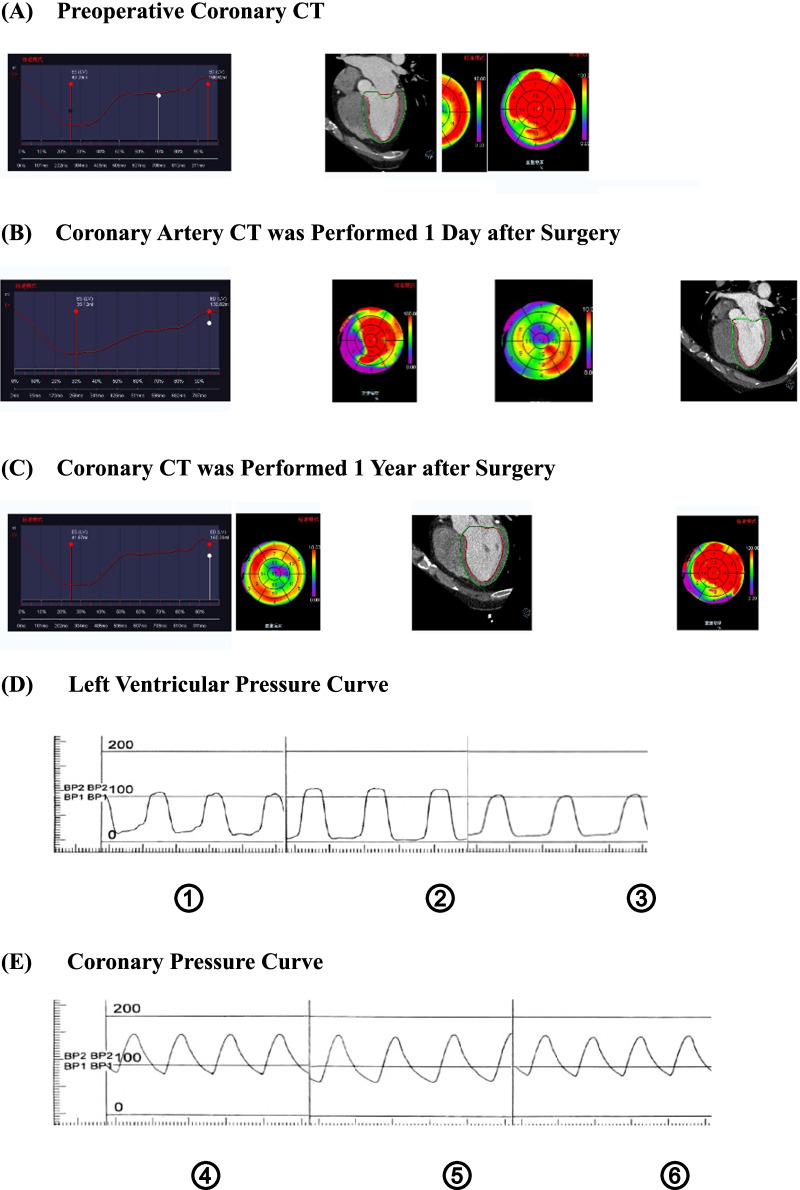
Comparison of coronary CT, left ventricular pressure profile and coronary pressure profile data before and after PCI in a patient with coronary artery disease. The patients treated with PCI intervention, whose left ventricular pressure as well as coronary pressure profiles were preserved and coronary CT was performed to obtain a more comprehensive data on the patient's left ventricular diastolic function. **a** Coronary Artery CT was performed Preoperatively. **b** Coronary Artery CT was Performed 1 Day after stent implantation. **c** Coronary Artery CT was Performed 1 Year after stent implantation. **d** Left ventricular pressure curve. ① represents pre-procedure period, ② represents the immediate post-procedure period, ③ represents one year after the procedure. **e** Coronary pressure curve. ④ represents pre-procedure period, ⑤ represents the immediate post-procedure period, ⑥ represents one year after the procedure

Pre-PCI, post-PCI coronary CT, left ventricular pressure profile and coronary pressure profile in patients with coronary artery disease are shown in Fig. 3. Although the pre-procedure coronary CT cut-off was at the maximum diastolic volume, it was still larger than the optimal diastolic volume at 1-day post-procedure. It suggested that there was a decrease in diastolic function in the short post-procedure period. The left ventricular pressure curve (Fig. 3d) showed that the left ventricular pressure was higher in the immediate post-procedure period than in the pre-procedure period, which means that diastolic function was reduced. One year after the procedure, the left ventricular pressure was measured again and showed an improvement in intraventricular pressure compared with the immediate post-procedure period and the pre-procedure period. Similarly, the coronary pressure curve (Fig. 3e) showed a consistent change with the LV pressure curve, which means that diastolic function improved 1 year after stent implantation compared with the immediate postoperative period and the preoperative period.

**Fig. 4:**
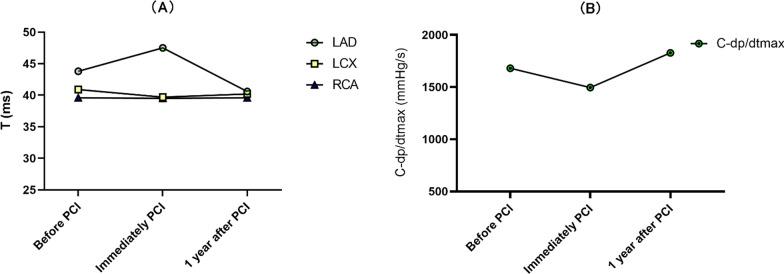
Single stent-LV diastolic function and coronary artery compliance. **a** We recorded T values from patients with LAD, LCX and RCA single stent implantation before, immediately after and one year after the procedure and used line graphs to visualize the relationship between T values and stenting site. **b** The maximum rate of coronary pressure drop in the immediate postoperative period in patients with LAD stenting was selected as an indicator of coronary artery compliance and thus compared to an indicator of left ventricular diastolic function

**Fig. 5:**
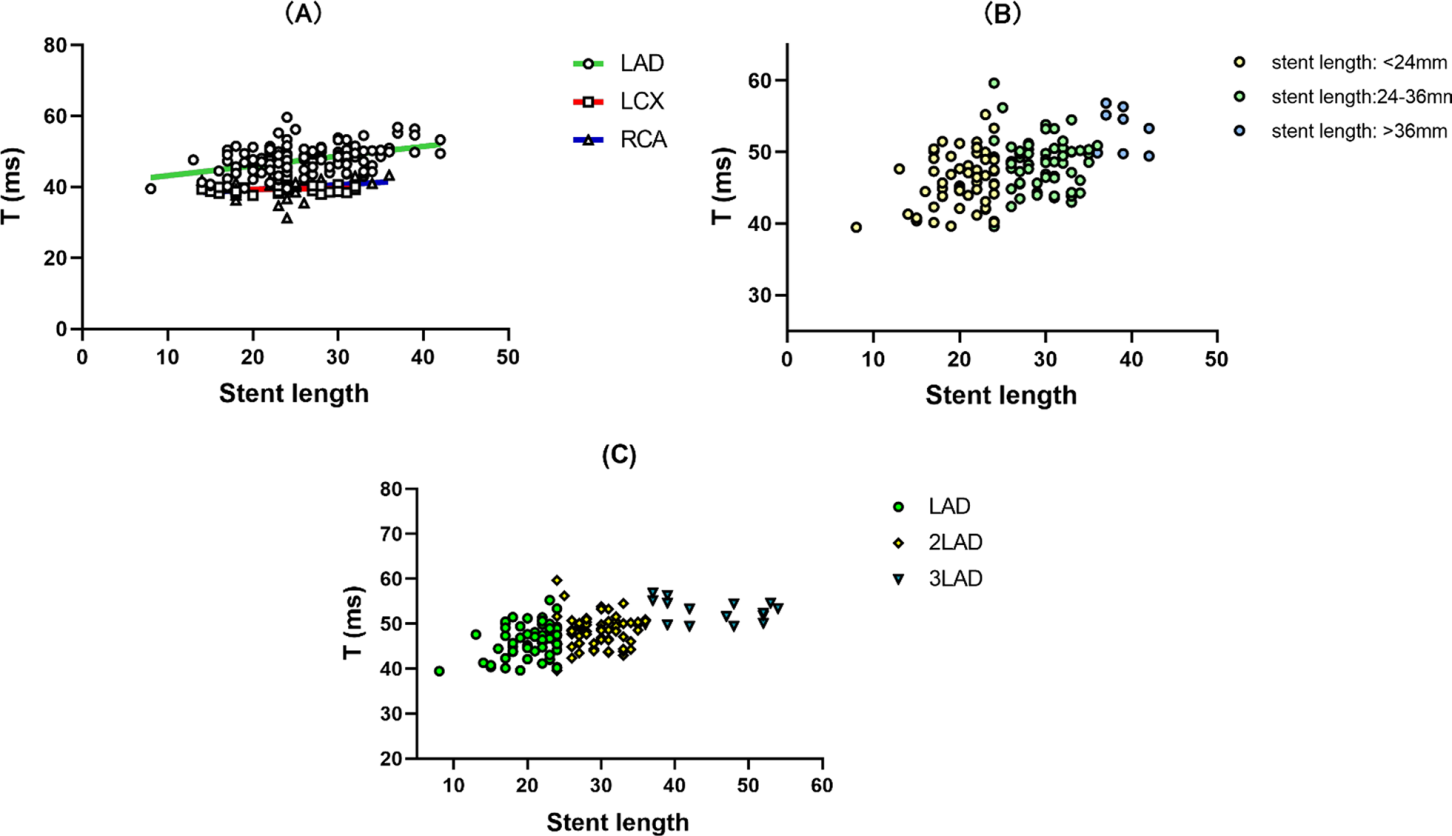
The correlation between T and stent length. **a** To investigate the relationship between stent length and T at different implantation sites and whether there are differences between implantation sites. And T-values are measured in the immediate post-operative period. **b** Patients with LAD stents were divided into three groups according to the length of the stent, using a cut-off of 24 mm and 36 mm, in order to visualize the change in T values between these groups. T-values are measured in the immediate post-operative period. **c** The total stent length was calculated for each patient with LAD implanted stent. Patients with LAD, 2LAD, and 3LAD were divided into three groups using a 24 mm and 36 mm stent length cut-off to visualize the relationship between T and stent length. T-values are measured in the immediate post-operative period

For patients with LAD stents, T values before, immediately after and 1 year after stent implantation showed a deterioration in diastolic function during the immediate postoperative period, but not for patients with LCX and RCA (Fig. 4a), and the maximum rate of decrease in intracoronary pressure as a proxy for coronary artery compliance in patients with LAD. It was found that coronary artery compliance was consistent with a decrease in LV diastolic function and coronary artery compliance in the immediate postoperative period (Fig. 4b). By grouping stent lengths, a correlation was found between T and stent length in patients with LAD implantation (Pearson R = 0.437, *P* < 0.01), whereas the correlation between T and stent length for patients with LCX and RCA implantation had no statistical significance (LCX: Pearson R = 0.206, *P* = 0.283, RCA: Pearson R = 0.246, *P* = 0.160) (Fig. 5a); after grouping the stent lengths of patients with LAD stenting, a one-way ANOVA revealed statistically significant differences in T values between stent length groups (F = 17.229, *P* < 0.001), and a two-way comparison between the LAD, LCX and RCA groups revealed that the differences in T values in groups I, II and III had statistical significance. The difference between the LAD, LCX and RCA groups was statistically significant (LSD: *P* < 0.01). The T value of the left ventricular diastolic function index increased with increasing stent length. The degree of deterioration of LVDF gradually increased (Fig. 5b).

**Fig. 6:**
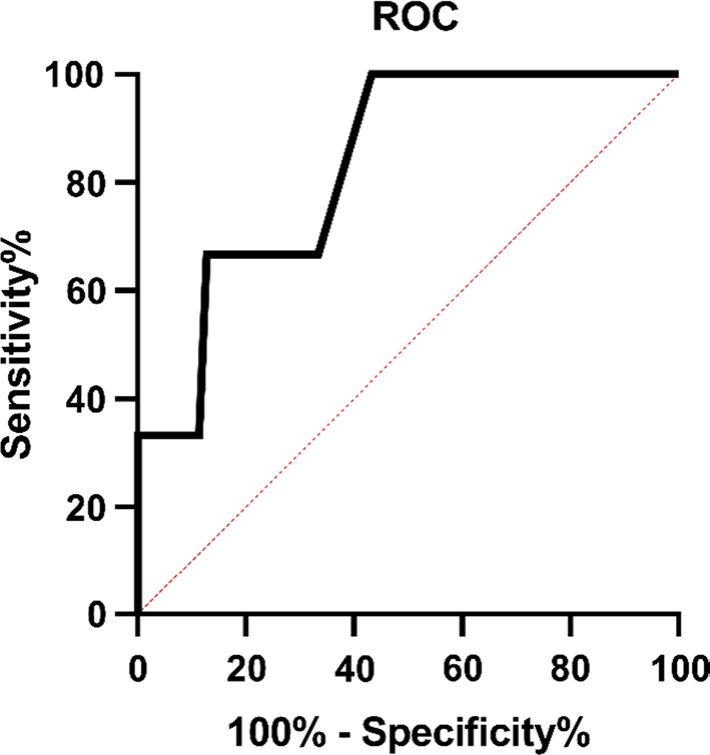
ROC curve. The upper limit of normal values of T was used as a cut-off for the group. And patients with LAD stents were divided into normal and abnormal groups. The T values of both groups were analysed by ROC curves to determine the threshold length of the stent that could cause abnormal T values in LAD implanted stents

Patients in group D were divided into groups, which were LAD single, two and three stent, according to the number of stents, with LAD two and three stents accounting for only 8.3% and 2.1% of the LAD implanted stents. A one-way ANOVA was applied to find that T values increased with the number of stents in each group, which means the differences in stent length and T-value were statistically significant in the different LAD groups (stent length F = 147.408, *P* < 0.001; T-value F = 11.834, *P* < 0.001) (Additional file 1: Table S2), and there was a correlation between the increasement in T-value and stent length (Pearson R = 0.546, *P* < 0.001). The correlation is that the longer the stent length is, the greater the increasement in LV diastolic function after PCI is. The longer the stent length, the worse the LV diastolic function after PCI (Fig. 5c). After grouping the T values in the LAD, 2LAD and 3LAD groups according to the upper limit of normal T values, a ROC curve was plotted to determine the optimal cut-off value of 24.5 mm for stent length (sensitivity 0.602, specificity 1.000, area under the curve 0.844, CI: 0.747–0.942, *p* < 0.001) (Fig. 6).

## Discussion

The data were collected and analyzed from 540 PCI patients and the data showed that, excluding other factors, the diastolic function index T increased with age, and the diastolic function of patients decreased (Pearson R = 0.696, *P* < 0.001). Further grouped patients undergoing PCI showed that diastolic function decreased significantly in the immediate post-PCI period in patients with LAD lesions, whereas there was no significant decrease in diastolic function in patients with LCX and RCA lesions. With the number of stents implanted increased, T values also increased. The total length of the stent implanted was positively correlated with the T value, which shows that the longer the length of the stent implanted is, the worse the patient's diastolic function in the immediate postoperative period is. But when coronary angiography was repeated 1 year after stent implantation, the T value of the patient's left ventricular diastolic function was found to have recovered.

Diastolic heart failure is a common clinical syndrome. The prevalence is increasing because of an ageing population and increasing co-morbidity burden. More than half of patients with exertional dyspnea of unknown origin assessed invasively have diastolic heart failure, and more than 70% of heart failure patients over 65 years old have diastolic heart failure [19–21]. Left ventricular diastolic function plays an important role in the evaluation of clinical symptoms, treatment options and prognosis of patients with cardiovascular disease, and early and aggressive treatment of patients with diastolic dysfunction can prevent or delay the onset of heart failure [22]. In this study, coronary atherosclerosis and the implantation of stents during PCI caused a decrease in the compliance of the coronary arteries that travel on the surface of the heart, and the small blood vessels that distribute between the myocardium, resulting in a constricting effect on the heart and thus affecting diastolic function.

Coronary atherosclerosis is caused by the accumulation of LDL to form lipid plaques, and the narrowing and narrowing of the lumen is accompanied by a hardening and loss of elasticity of the vessel wall. The degree of coronary artery disease has been shown to correlate with left ventricular diastolic function [6, 23, 24]. A foreign clinical study investigated the relationship between the degree of coronary stenosis and left ventricular diastole by using non-invasive coronary CT, with an increase in LVEDP of 0.8 mmHg for every 0.05–1.1 increase in coronary segmental stenosis score [7], suggesting a decrease in left ventricular compliance due to myocardial ischemia because of insufficient coronary blood supply. In this study, the degree of coronary artery disease was found to be involved in diastolic insufficiency through myocardial ischemia. The results show that coronary artery disease and the changes in ischemia and hypoxia in the small vessels it affects can cause a decreasement in diastolic function, and that the more severe the coronary artery disease is, the worse the diastolic function is. And coronary atherosclerosis may reduce the compliance of the coronary arteries and thus have a restraining effect on the contraction and diastole of the heart. Atherosclerosis of the coronary arteries can reduce the elasticity of the vessels and have a constricting effect on the heart, theoretically resulting in a reduction in diastolic function. It was found by this study that the change in LV diastolic function was mainly affected by LAD and its small vessel lesions. And what is more, the more severe the LAD lesion is, the worse the diastolic function in the absence of external intervention is. One year after stent implantation, the patient's left ventricular function basically returned to normal after one year as the stent opened the vessel and as the coronary vessels adapted to the stent allowing vascular compliance to return, thus restoring the blood and oxygen supply to the ventricles.

Previous studies have done some comparison on LVEF for patients undergoing PCI using echocardiography before, 1 day after and 3–6 months after the procedure. It was found that LVEF improved significantly at 1 day and 3–6 months after the procedure, whereas diastolic function improved at 1 day but did not change 3–6 months after the procedure [25]. Other studies have shown that LV diastolic function is altered after PCI [26–28]. These studies have used non-invasive tests and have small sample sizes. The present studies used the left ventricular isovolumic relaxation time constant T, which is less affected by other factors, to represent left ventricular diastolic function using an invasive catheter method and therefore, it could be more accurately to reflect the changes in left ventricular diastolic function for patients after stent implantation [13]. In this study, the effect of stent placement site and stent length on left ventricular diastolic function was further investigated. It was shown that as longer LAD stent lengths were inserted, lower left ventricular diastolic function was observed. The study also found a significant decrease in coronary artery compliance after LAD stenting, so it is reasonable to assume that the decrease in diastolic function may be caused by an effect on coronary artery compliance. Of these studies, further research is needed to determine whether the left ventricular coronary artery activates a mechanism that affects its compliance after coronary stenting.

This study had some limitations. First, the patients selected were limited to Fengxian District Central Hospital and not randomly selected patients across the country; second, the study was retrospective and not prospective. Therefore, the effect of stent length and stent implantation site on post-PCI needs to be further investigated.

## Conclusion

In this study, by comparing patients with different degrees of coronary artery stenosis, it was verified that coronary atherosclerosis can lead to a decrease in compliance of the coronary arteries travelling on the surface of the heart and the small vessels distributed between the myocardium, and it was also found that as the degree of coronary stenosis increased, LV diastolic function gradually decreased; and by further investigation, it was found that the degree of LAD vessel lesion and the LAD stent implantation were closely related to the degree of reduction in LV diastolic function. The discovery could provide new clinical ideas for active intervention in postoperative patients with cardiovascular events such as diastolic insufficiency, and also provide some clinical evidence to help clinicians better judge the application of stents in patients who need to undergo PCI length.

## Supplementary Information

**Additional file 1: Fig. S1**. The figure S1 is a supplemental data to Fig. 2. The five graphs represent the LV pressure profiles for each group of patients. The figure S1(A) represents the first subgroup of the control group, the figure S1(B) represents the second subgroup of the control group, the figure S1(C) represents the third subgroup of the control group, the figure S1(D, E) represents the fourth subgroup of the control group. **Table S1** Single stent—left ventricular diastolic function data. To investigate the effect of stenting site on left ventricular diastolic function. According to the stent implantation site, patients implanted with a single stent were grouped and compared their LV diastolic function preoperatively, immediately postoperatively and one year postoperatively. **Table S2** LAD single stent vs LAD multi-stent immediate post-operative data. To investigate whether T values are related to the number of stents implanted by comparing LV diastolic function indices in the immediate postoperative period in single versus multi-stent patients with LAD implantation.

## Data Availability

The data that support the findings of this study are not openly available due to human data and are available from the corresponding author upon reasonable request.

## Abbreviations

LVDF: Left ventricular diastolic function
CHD: Coronary heart disease
LAD: Left anterior descending artery
LCX: Left circumflex artery
RCA: Right coronary artery
LVDD: Left ventricular diastolic dysfunction
PCI: Percutaneous coronary intervention
NYHA: New York Heart Association
K: Left ventricular stiffness index
DPV: Diastolic-pressure–volume curve
CEDV: End-diastolic coronary volume
CESV: End-systolic coronary volume
C + dp/dtmax: Maximum rate of increase in coronary pressure
C-dp/dtmax: Maximum rate of decrease in coronary pressure
CAG: Coronary angiography
LVEDV: Left ventricular end-diastolic volume
LVESV: Left ventricular end-systolic volume
LVEF: Left ventricular ejection fraction
LV+dp/dtmax: Maximum rate of left ventricular pressure rise
LV-dp/dtmax: Maximum rate of left ventricular pressure fall
PFR: Maximum left ventricular filling rate
T: Time constant of relaxation
