# Supplementary material for: The constricting effect of reduced coronary artery compliance on the left ventricle is an important cause of reduced diastolic function in patients with coronary heart disease
Source: BMC Cardiovasc Disord. 2022 Aug 17;22:375. doi: 10.1186/s12872-022-02809-0 (PMC9382726; doi:10.1186/s12872-022-02809-0)
Supplement: Supplementary file 1 — Additional file 1: Fig. S1. The figure S1 is a supplemental data to Fig. 2. The five graphs represent the LV pressure profiles for each group of patients. The figure S1(A) represents the first subgroup of the control group, the figure S1(B) represents the second subgroup of the control group, the figure S1(C) represents the third subgroup of the control group, the figure S1(D, E) represents the fourth subgroup of the control group. Table S1 Single stent—left ventricular diastolic function data. To investigate the effect of stenting site on left ventricular diastolic function. According to the stent implantation site, patients implanted with a single stent were grouped and compared their LV diastolic function preoperatively, immediately postoperatively and one year postoperatively. Table S2 LAD single stent vs LAD multi-stent immediate post-operative data. To investigate whether T values are related to the number of stents implanted by comparing LV diastolic function indices in the immediate postoperative period in single versus multi-stent patients with LAD implantation. [file 12872_2022_2809_MOESM1_ESM.docx]

**Supplemental Material**

**Figure S1**

**
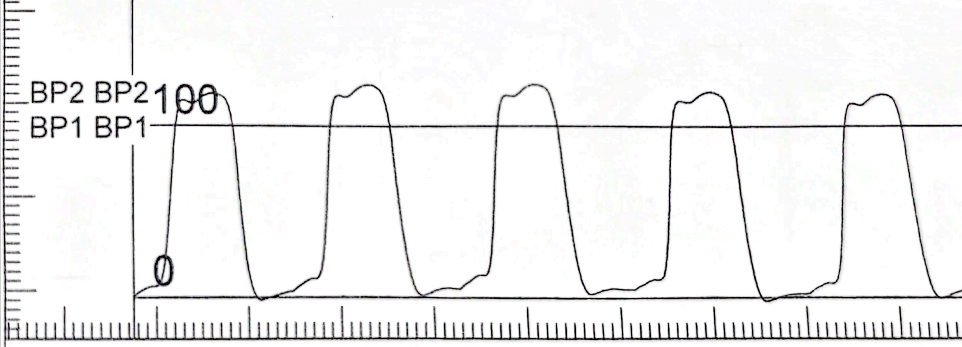
(A) 45-years-old; male; T:25.21**

**(B) 60-years-old; male; T:31.48**

**
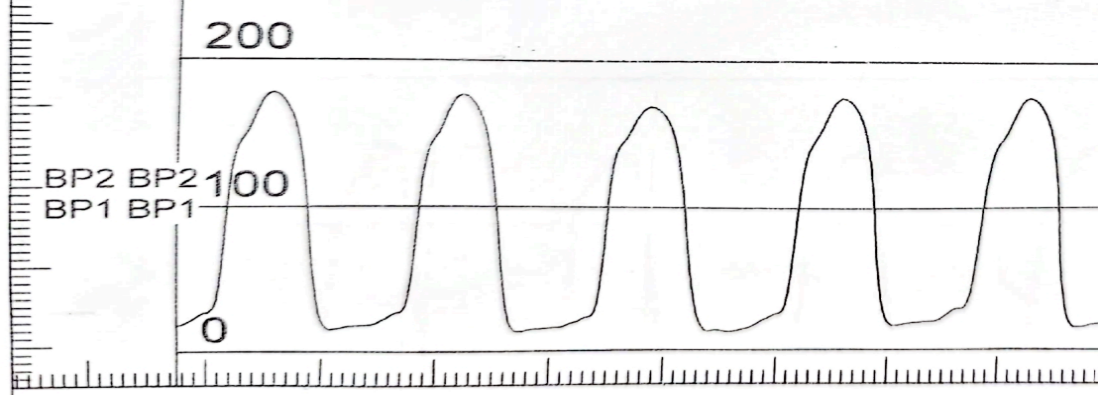
**

**(C) 66-years-old; male; T:31.93**

**
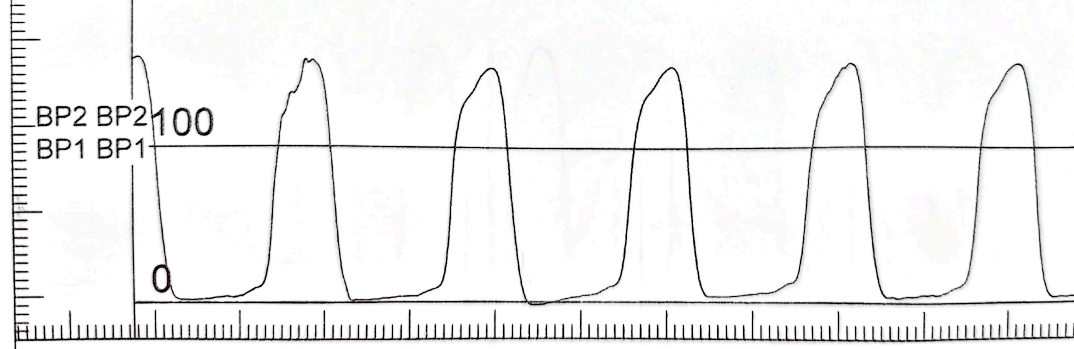
**

**(D) 76-years-old; male; T:35.63**

**
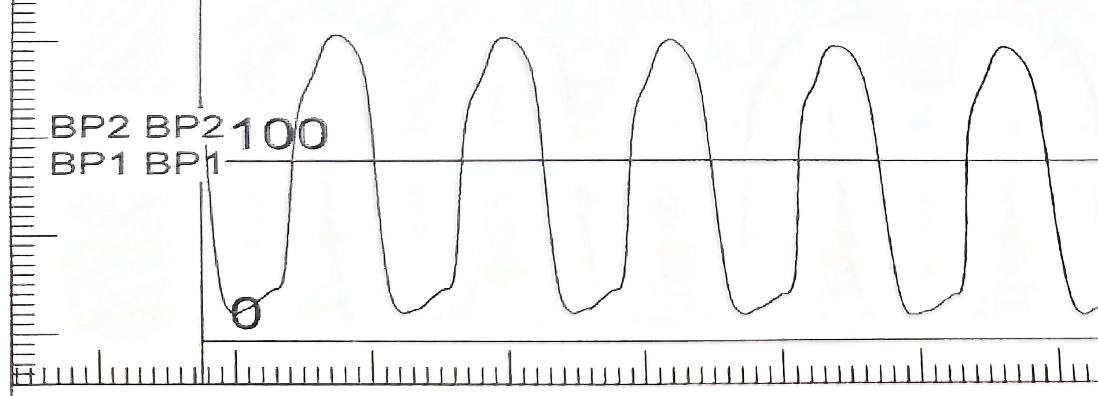
**

**(E) 85-years-old; male; T:47.04**

**
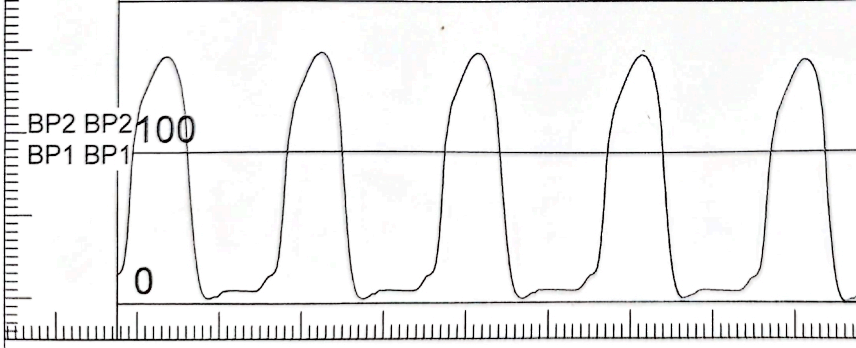
**

| **Table S1. LAD single stent - left ventricular diastolic function data** | | | | |
| --- | --- | --- | --- | --- |
|  | LAD | LCX | RCA | P value |
|  | (n=129) | ( n=29 ) | ( n=34 ) |  |
| **Pre-PCI** | | | | |
| PFR | 198.3(8.8)^*^ | 225.4(11.2) | 225.2(12.0) | <0.001 |
| T | 43.8(3.7)^*^ | 39.6(0.9) | 40.9(3.7) | <0.001 |
| K | 4.9(0.6)^*^ | 4.3(0.2) | 4.1(0.2) | <0.001 |
| LV+dp/dtmax | 2305.7  (2257.1-2364.4)^*^ | 2012.5  (2005.2-2027.1) | 2005.3  (1973.0-2049.5) | <0.001 |
| LV-dp/dtmax | 2207.5  (2166.6-2247.5) ^*^ | 1899.0  (1888.7-1911.6) | 1903.1  (1893.7-1919.2) | <0.001 |
| CEDV | 3.8(0.1)^*^ | 4.2(0.1) | 4.1(0.1) | <0.001 |
| CESV | 3.1(0.2) | 3.1(0.1) | 3.1(0.1) | 0.062 |
| C+dp/dtmax | 1941.4(360.7) | 2089.2(104.7) | 2077.4(65.7) | 0.005 |
| C-dp/dtmax | 1680.1(250.7) | 1753.1(81.1) | 1749.1(54.1) | 0.163 |
| **IM-post-PCI** | | | | |
| Stent length | 25.9(6.4) | 24.5(5.7) | 23.6(4.7) | 0.168 |
| PFR | 183.1(23.1)^*^ | 226.0(11.0) | 225.8(12.5) | <0.001 |
| T | 47.5(4.0)^*^ | 39.5(1.1) | 39.7(2.6) | <0.001 |
| K | 5.9(0.2)^*^ | 4.2(0.3) | 4.1(0.1) | <0.001 |
| LV+dp/dtmax | 2477.0  (2446.4-2535.4)^*^ | 2026.2  (1998.9-2088.0) | 2010.7  (1972.4-2054.6) | <0.001 |
| LV-dp/dtmax | 2295.6  (2243.9-2348.5)^*^ | 1903.5  (1890.9-1921.7) | 1906.0  (1891.1-1930.8) | <0.001 |
| CEDV | 3.4(0.1)^*^ | 4.2(0.1) | 4.2(0.1) | <0.001 |
| CESV | 2.7(0.3)^*^ | 3.1(0.1) | 3.1(0.1) | <0.001 |
| C+dp/dtmax | 1747.7(365.6)^*^ | 2087.2(111.5) | 2068.1(92.9) | <0.001 |
| C-dp/dtmax | 1495.0(259.9)^*^ | 1753.9(82.4) | 1747.9(53.0) | <0.001 |
| **Post-PCI** | | | | |
| PFR | 222.5(18.3) | 225.4(11.2) | 225.2(12.0) | 0.986 |
| T | 40.6(4.2) | 39.6(1.7) | 40.2(1.7) | 0.479 |
| K | 4.2(0.6) | 4.2(0.2) | 4.1(0.2) | 0.113 |
| LV+dp/dtmax | 2075.0  (1881.9-2225.4) | 2068.5  (1974.9-2117.7) | 2010.5  (1969.5-2059.7) | 0.252 |
| LV-dp/dtmax | 1872.6  (1731.0-2019.3) | 1897.4  (1834.6-1928.5) | 1899.0  (1864.7-1958.1) | 0.673 |
| CEDV | 4.1(0.1) | 4.1(0.3) | 4.1(0.4) | 0.831 |
| CESV | 3.1(0.4) | 3.1(0.3) | 3.1(0.4) | 0.906 |
| C+dp/dtmax | 2097.0(311.7) | 2091.4(134.6) | 2080.9(118.7) | 0.874 |
| C-dp/dtmax | 1828.7(224.2) | 1756.2(151.8) | 1765.8(120.0) | 0.121 |
| Values are mean±standard deviation or median (interquartile spacing). K, stiffness index; LV+dp/dtmax, maximum rate of left ventricular pressure rise; LV-dp/dtmax, maximum rate of left ventricular pressure drop; CEDV, coronary artery end-diastolic volume; CESV, coronary artery end-systolic volume; C+dp/dtmax, maximum rate of coronary artery pressure rise; C-dp/dtmax, maximum rate of coronary artery pressure drop. '*'indicates that the difference is statistically significant in the same row. | | | | |

| **Table S2. LAD single stent vs LAD multi-stent immediate post-operative data** | | | | |
| --- | --- | --- | --- | --- |
|  | LAD | 2LAD | 3LAD | P value |
|  | ( n=129 ) | ( n=12 ) | ( n=3 ) |  |
| Stent length | 25.9(6.4)^a^ | 47.8(11.6)^b^ | 83.3(6.7)^c^ | <0.001 |
| PFR | 183.1(23.1) | 186.3(22.2) | 183.0(29.3) | 0.900 |
| T | 47.6(3.8)^a^ | 51.7(2.7)^b^ | 55.5(1.7)^b^ | <0.001 |
| K | 5.9(0.2)^a^ | 6.2(0.1)^b^ | 6.2(0.1)^b^ | <0.001 |
| LV+dp/dtmax | 2477.0  (2446.4-2535.4) | 2486.6  (2449.0-2554.7) | 2463.7  (2437.1-2526.4) | 0.698 |
| LV-dp/dtmax | 2295.6  (2243.9-2348.5) | 2339.0  (2314.4-2381.6) | 2285.9  (2275.3-2338.4) | 0.115 |
| CEDV | 3.4(0.2)^a^ | 3.7(0.1)^b^ | 3.7(0.1)^b^ | <0.001 |
| CESV | 2.7(0.3) | 2.8(0.2) | 2.7(0.1) | 0.857 |
| Values are mean±standard deviation or median (interquartile spacing). K, stiffness index; LV+dp/dtmax, maximum rate of left ventricular pressure rise; LV-dp/dtmax, maximum rate of left ventricular pressure drop; CEDV, coronary artery end-diastolic volume; CESV, coronary artery end-systolic volume; C+dp/dtmax, maximum rate of coronary artery pressure rise; C-dp/dtmax, maximum rate of coronary artery pressure drop. Each subscript letter denotes a subset of categories whose row proportions do not differ significantly from each other at the .05 level. | | | | |
